# mHealth app usage amongst paediatric department doctors in South Africa

**DOI:** 10.4314/ahs.v23i3.24

**Published:** 2023-09

**Authors:** Shahid Mahmood, Ashraf Coovadia, Abdullah E Laher, Ahmed Adam

**Affiliations:** 1 University of the Witwatersrand Faculty of Health Sciences, Paediatrics, South Africa; 2 University of the Witwatersrand Faculty of Health Sciences, Emergency Medicine, South Africa; 3 University of the Witwatersrand Faculty of Health Sciences, Urology, South Africa

**Keywords:** Medical apps, mHealth, mobile health, smartphone health, information technology, drug dosing app, medical diagnostic app

## Abstract

**Background:**

Smartphone and mobile health (mHealth) applications (apps) have become an integral part of the day-to-day function of healthcare professionals, allowing quick, comprehensive, and up-to-date access to current clinical guidelines and other reference material.

**Objective:**

To evaluate the extent and nature of use of mHealth apps by paediatric department doctors in South Africa.

**Methods:**

E-mails requesting study participation were sent out to 285 paediatric department doctors employed at six hospitals affiliated to the University of the Witwatersrand. Willing participants were directed to complete the online study questionnaire.

**Results:**

A total of 150 respondents completed the questionnaire. All respondents owned a mobile device and already had one or more mHealth apps, 95.3% were unaware of any regulatory body responsible for regulating the use of mHealth apps, 86.0% did not have access to free Wi-Fi at work and 87.3% used an mHealth app at least once daily. Drug dosing (81.3%), diagnostic (59.3%) and clinical decision-making (44.7%) apps were the most common app categories with Medscape® (62.0%) and EMGuidance® (41.3%) being the most frequently used apps. Peer recommendation (76.0%), app credibility (74.0%) and app functionality (66.0%) were the most common factors that were considered by respondents prior to downloading or using an mHealth app.

**Conclusion:**

Medical apps are frequently used among paediatric medical doctors of all ranks. Drug dosing, diagnostic and clinical decision-making apps are the most common app categories in use. Improved awareness of the regulations pertaining to the use of mHealth apps amongst doctors is required.

## Introduction

In the past few decades, advancements in information and mobile technology have led healthcare professionals accessing medical information to move from desktops computers and laptops to handheld devices and the use of smartphones.^1^ Mobile health (mHealth) applications (apps) have been used to guide medical practitioners in diagnosis^2^ and drug prescription.^3^ Apart from healthcare practitioners, mHealth apps have also been used to assist with patient self-management.^4^

With the high levels of smartphone ownership and a surge in the growth of mHealth apps, smartphones have now become an integral part of the day-to-day function of healthcare professionals,^5^ allowing quick, comprehensive, and up-to-date access to current clinical guidelines and other reference material.^6^

Apps are software programs that have been developed to run on a computer or mobile device to accomplish a specific purpose.^7^ Faster processors, improved memory, smaller batteries, and highly efficient operating systems have led to the development of a large number of medical apps for both professional and personal use.^8^ It is estimated that more than 50% of global smartphone users have downloaded an mHealth app.^9^ In just the second quarter of 2021, there was a 53% surge in usage of medical and health related mobile apps in the USA.^10^

Healthcare professionals can use medical apps to accomplish many vital tasks including access to patient health records, communication, patient consultation and management, access to updated medical information and educational resources, clinical decision making and medical training.^11^ Despite the numerous benefits associated with the use of mHealth apps, there are concerns regarding their reliability, ability to protect personal data and privacy, impact on the doctor-patient relationship, proper integration into the workplace, standards and accuracy of content, and medico-legal and ethical implications.^6^,^12^–^15^ Although there are regulations governing the manufacture and use of mHealth apps internationally,^16^,^17^ there is a lack of clear regulations pertaining to the use of mHealth apps in South Africa.^18^

Majority of digital health apps have been developed in Europe and the United States with Africa and South America contributing a very small proportion.^9^ A systematic review that evaluated mHealth apps implemented in sub-Saharan Africa between 2006 and 2016 reported that only 487 mHealth apps were developed in the region,^19^ which is small fraction of the estimated number of mHealth apps available worldwide.^20^ A survey conducted among 50 emergency medicine consultants and registrars in South Africa between December 2015 and February 2016 reported that 92% of respondents had one or more mHealth apps on their mobile device, of whom 67% used one or more of these apps daily.^21^

There has been a steady increase in the number of mobile internet users in South Africa. In 2021, there were 36.45 million mobile internet users and by 2026, it is projected that there will be 42.82 million mobile internet users in South Africa.^22^ Despite, the increasing utilization of mHealth apps in South Africa by both health care practitioners and the general public,^23^,^24^ there is a paucity of data pertaining to the frequency and characteristics of mHealth app use in South Africa. Hence, the aim of this study was to evaluate the extent and nature of mHealth apps use by paediatric department doctors at six hospitals that are linked to the University of the Witwatersrand in Johannesburg, South Africa.

## Methods

The study entailed a prospective cross-sectional questionnaire-based design. The study was conducted amongst paediatric department doctors working at three central (Rahima Moosa Mother and Child Hospital (RMMCH), Chris Hani Baragwanath Academic Hospital (CHBAH) and Charlotte Maxeke Johannesburg Academic Hospital (CMJAH)) and three satellite (Thelle Mogoerane Hospital, Sebokeng Hospital and Klerksdorp Hospital) hospitals. Permission to conduct the study was obtained from the clinical manager of the respective hospitals, while ethics clearance was obtained from the Human Research Ethics Committee of the University of Witwatersrand (certificate no. M171141).

At the time that the study was conducted, a total of 285 doctors were employed at the respective paediatric departments of the six included hospitals. Emails requesting study participation were sent out to all 285 doctors, with reminder e-mails being sent to non-responders every three to four weeks over the period of data collection. Each e-mail included information pertaining to the study aim and objectives and a link directing the potential participant to the study questionnaire. The e-mail also included a statement indicating that study consent will be automatically assumed if the survey was completed.

Data was collected between 01 February and 31 August 2018. The study questionnaire was adapted from a questionnaire that was used by Payne et al., who conducted a study investigating smartphone and medial app use among medical student and junior doctors in the United Kingdom (UK).^25^ Questions included in the questionnaire pertained to participant rank/position, frequency of medical app usage, categories of medical apps used, factors taken into consideration prior to downloading or using a medical app, criteria for recognizing a high-quality app and commonly used medical apps.

Collected data was thereafter entered into Microsoft Excel® (Microsoft 365, Version 16.0.13029.20232) and subsequently exported to Stata version 16 (StataCorp Limited, Texas, United States of America) for statistical analysis. Respondents were categorized into four groups based on experience (consultants, registrars, medical officers, and interns). Since all data were categorical in nature, these were described using frequency and percentage tables and graphs. Data pertaining to the frequency of medical app usage, the categories of medical apps used, and the factors taken into consideration prior to downloading or using a medical app were compared between the four groups using either the Pearson's chi-squared test (≥5 variables in any field) or the Fisher exact test (<5 variables in any field). The level of significance was set at p=0.05.

## Results

Of the 285 doctors to whom e mail/s were sent requesting study participation, a total of 150 completed the online survey, giving a response rate of 52.6%. Majority of the respondents were registrars (n=61, 40.7%), followed by consultants (n=49, 32.7%), interns (n=22, 14.7%) and medical officers (n=18, 12.0%). All 150 respondents owned a mobile device and used one or more mHealth apps. Most of the participants were unaware of any regulatory body that is responsible for regulating the use of mHealth apps (n=143, 95.3%) and most did not have access to free Wi-Fi at work (n=129, 86.0%). Table 1 describes the frequency of mHealth app usage, the categories of mHealth apps used, and the factors taken into consideration prior to downloading or using an mHealth app.

**Table 1:** Frequency of mHealth app usage, categories of mHealth apps used, and factors taken into consideration prior to downloading or using an mHealth app

	Total (n=150)	Consultants (n=49)	Registrars (n=61)	Medical Officers (n=18)	Interns (n=22)	p-value
**Frequency of mHealth** **app usage**						
At least once daily	131 (87.3)	39 (79.6)	53 (86.9)	18 (100)	21 (95.5)	^a^ 0.031
Not everyday	19 (12.7)	10 (20.4)	8 (13.1)	0 (0)	1 (4.5)	
**Categories of mHealth** **apps used**						
Drug dosing tools	122 (81.3)	35 (71.4)	53 (86.9)	18 (100)	16 (72.7)	^b^ 0.024
Diagnostic tools	89 (59.3)	28 (57.1)	34 (55.7)	10 (55.6)	17 (77.3)	0.325
Clinical decision-making tools	67 (44.7)	18 (36.7)	28 (45.9)	8 (44.4)	13 (59.1)	0.153
Secure messaging	46 (30.7)	16 (32.6)	17 (27.9)	6 (33.3)	7 (31.8)	0.942
Electronic health records	37 (24.7)	13 (26.5)	15 (24.6)	5 (27.8)	4 (18.2)	0.877
Note keeping and documentation tools	27 (18.0)	11 (22.4)	9 (14.7)	4 (22.2)	3 (13.6)	0.608
Patient engagement	14 (9.3)	8 (16.3)	4 (6.6)	2 (11.1)	0 (0)	0.013
**Factors considered** **prior to downloading** **or using an mHealth** **app**						
Peer recommendation	114 (76.0)	33 (67.3)	45 (73.8)	17 (94.4)	19 (86.4)	0.075
App credibility	111 (74.0)	32 (65.3)	47 (77.0)	15 (83.3)	17 (77.3)	0.403
App functionality	99 (66.0)	33 (67.3)	39 (63.9)	14 (77.8)	13 (59.1)	0.639
App usability	96 (64.0)	35 (71.4)	35 (57.4)	12 (66.7)	14 (63.6)	0.495
Used or recommended by a senior	89 (59.3)	17 (34.7)	40 (65.6)	15 (83.3)	17 (77.3)	^c^ <0.001
Cost of the app	88 (58.7)	33 (67.3)	33 (54.1)	10 (55.6)	12 (54.5)	0.064
Internet/ data availability	86 (57.3)	32 (65.3)	37 (60.7)	9 (50.0)	8 (36.3)	0.116
Clinical impact	75 (50.0)	32 (65.3)	26 (42.6)	10 (55.6)	11 (50.0)	0.125
Privacy and security	39 (26.0)	16 (32.6)	14 (23.0)	4 (22.2)	5 (22.7)	0.799

a consultants vs medical officers (p=0.019)

b consultants vs registrars (p=0.044); consultants vs medical officers (p=0.007)

c consultants vs registrars (p=0.001); consultants vs medical officers (p<0.001); consultants vs interns (p<0.001)

With regards to the frequency of mHealth app usage, most respondents (n=131, 87.3%) reported that they used an mHealth apps almost daily, with there being a significantly higher rate of usage among medical officers compared to consultants (p=0.019). There were no other statistically significant differences between groups.

Drug dosing tools (n=122, 81.3%), followed by diagnostic tools (n=89, 59.3%) and clinical decision-making support tools (n=67, 44.7%) were the most common categories of mHealth apps used by the respondents. Compared to consultants, registrars (p=0.004) and medical officers (p=0.007) were significantly more likely to use drug dosing tools. There were no other statistically significant differences between groups with regards to the other categories of mHealth apps used by the respondents.

Peer recommendation (n=114, 76.0%), followed by app credibility (n=111, 74.0%), app functionality (n=99, 66.0%) and app usability (n=96, 64.0%) were the most common factors that were considered by respondents prior to downloading or using an mHealth app. Compared to consultants, a significantly higher proportion of registrars (p=0.001), medical officers (p<0.001) and interns (p<0.001) reported that that were more likely to download or use an mHealth app if it was used or recommended by a senior. There were no other statistically significant differences between groups with regards to factors that were considered by respondents prior to downloading or using an mHealth app.

**Figure 1:**
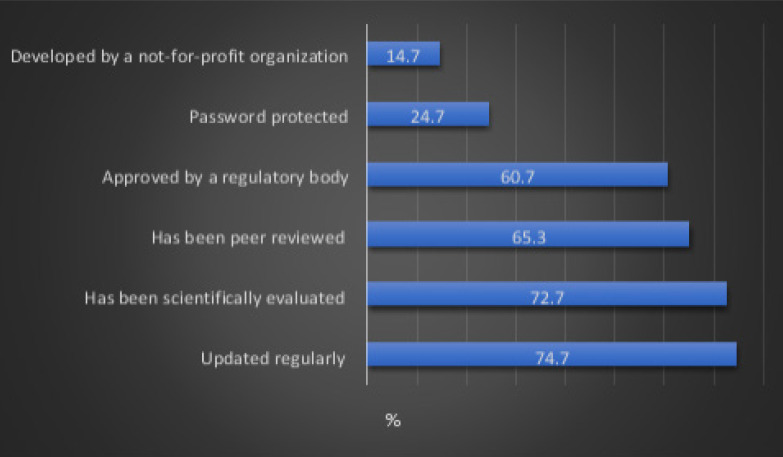
Criteria for recognizing a high-quality app and percentage of respondents that reported each of these criteria

Figure 1 describes the criteria for recognizing a high-quality app and the percentage of respondents that reported each of these criteria. Overall, the most reported criteria were apps that are updated regularly (n=112, 74.7%), apps that are scientifically evaluated (n=109, 72.7%) and apps that are peer reviewed (n=98, 65.3%).

**Figure 2:**
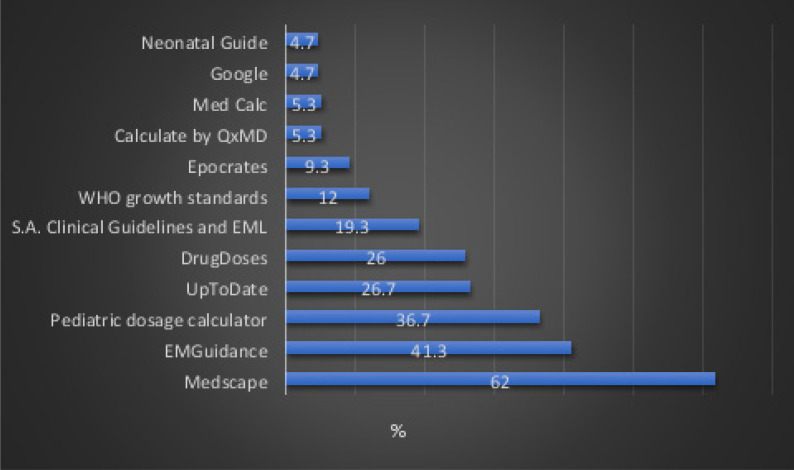
Frequently used apps among respondents

MHealth apps that are frequently used and the percentage of respondents that use each of these apps are described in figure 2. Medscape® (n=93, 62.0%), EMGuidance® (n=62, 41.3%) and Pediatric dosage calculator® (n=55, 36.7%) were the most frequently used apps.

## Discussion

To our knowledge, this is the first study to have evaluated the extent and nature of mHealth app use by paediatric doctors in sub-Saharan Africa. The increased reliance of healthcare professionals on electronic resources was identified in the 2012 Manhattan Research/Google Physician Channel Adoption study which showed that 87% of physicians used a smartphone or tablet in the workplace and that physicians spent twice as much time using online compared to print resources.^26^

In this study, all respondents reported using mHealth apps, while 87.3% reported usage at least once daily. Comparatively, A study conducted in 2012 comprising 504 medical students and junior doctors in the UK reported that 30% of respondents used an mHealth app at least once daily. Another study that was published in 2018 and comprised 300 physicians from Saudi Arabia reported that 53% of respondents used an mHealth app at least once daily. A further study that was published in 2019 and comprised 1014 general practitioners in Australia reported that 64% of respondents used mHealth related apps.^27^ In a more recent study published in 2021 comprising 198 doctors working in paediatric emergency care in the UK and Ireland, the authors reported that 86% of respondents used medical related apps on their mobile device, 89% used their device for web access daily, and 47% used formulary apps daily.^28^

In this study, although less experienced doctors (registrars, medical officers, and interns) used their mHealth apps more frequently than more experienced doctors (consultants), the difference in usage was only statistically significant between consultants and medical officers. Other studies have also reported that older doctors are less likely to adopt the use of mHealth apps compared to younger doctors.^29^,^30^ However, the influence of age on the usage of mobile applications is not entirely clear.^31^ A study showed that there was no direct relationship between age and mobile app use.^32^ Furthermore, the Manhattan Research/Google Physician Channel Adoption study reported that physicians who were ≥55 years spent nine hours per week using online resources related to their profession and that 64% of their overall online time was spent on resources pertaining to clinical decisions.^26^

There are currently more than 350 000 mHealth apps available globally that provide comprehensive and around the clock resources relating to evidence-based medicine.20 In this study, drug dosing tools (81.3%), diagnostic tools (59.3%) and clinical decision-making tools (44.7%) were the three most used mHealth apps in clinical practice, with Medscape® (62.0%) and EMGuidance® (41.3%) being the most frequently used apps. Medscape® has been ranked as one of the top mHealth apps with more than five million downloads and a rating of 4.5/5. It is used by approximately half of all doctors in the United States.^33^ It is not surprising that EMGuidance® was one of the most popular apps, as it was developed in South Africa and includes a formulary of medications and doses, including generic brands and other formulations that are sold in South Africa. It has more than 100 000 downloads with a rating of 4.8/5.^34^ in a previous study that was also conducted in South Africa, EMGuidance® was the most frequently used app that was used by 40% of respondents.^21^

In this study, peer recommendation (76.0%), app credibility (74.0%), app functionality (66.0%), and app usability (64.0%) were the common factors that were considered prior to downloading or using an mHealth app. A study pertaining to concerns of users of mHealth apps reported that usability, certifiability, safety, trust ability and security were the most important concerns identified by the study.^35^ App users should also ensure that the app has been validated prior to downloading and using the app. Although only 26% of respondents in our study reported privacy and security as a concern, a study by Plachkinova et al., identified significant privacy and security concerns among 38 mHealth apps that were evaluated in their study.^36^

Regarding data protection, privacy and other security concerns of mHealth apps developed in South Africa, there are various statutes that governs the development of digital health software. These include the Promotion of Access to Information Act (PAIA) 2 of 2000.^37^ the National Health Act 61 of 2003,^38^ the Health Professions Amendment Act 29 of 2007,^39^ the Protection of Personal Information Act (POPIA) 4 of 2013^40^ and the Medicines and Related Substances Amendment Act 14 of 2015.^41^ These statutes assist to provide a secure platform for messaging amongst health care practitioners, electronic health records data capturing and patient engagement.

In this study, the most common criteria reported for recognizing a high-quality mHealth app were app updated regularly (74.7%), scientifically evaluated (72.7%), peer-reviewed (65.3%) and approved by a regulatory body (60.7%). A systematic review that included 23 articles pertaining to the criteria for assessing the quality of mHealth apps identified seven main classes of assessment criteria which included design, content, usability, functionality, ethical considerations, security and privacy and user perceived value.^42^ Furthermore, Stoyanov et al., developed the end-user Mobile App Rating Scale (uMARS), which is a 20-item scale that was developed to provide end-users with a reliable method of assessing the quality of mHealth apps.^43^

The overall benefits of mHealth app usage in clinical practice is undisputed. However, due to resource constraints that are prevalent in low-middle income settings, clinicians are not always able to take full benefit of these resources. To allow for growth, and advancement of technology in low middle income countries (LMIC), efforts should be channelled at improving internet access and providing access to smart devices in medical facilities. It is also imperative to educate junior staff regarding the use of validated and evidence-based apps.

There are some limitations to this study. Since participation was voluntary and the study questionnaire was distributed electronically, this could have resulted in a selection bias where respondents may perhaps have been more technologically literate than non-responders. Also, our questionnaire did not explore items pertaining to the number of mHealth apps in use, the amount of daily time spent, dependence and perceptions pertaining to the use of mHealth apps. Another limitation is that approximately half the number of doctors who were invited to participate in the study did not complete the questionnaire. Despite these limitations, data obtained from this study provides a base for further studies.

## Conclusion

mHealth apps were frequently used by all ranks of doctors that participated in this study, which is in keeping with a rising trend of mHealth app use in clinical practice globally. The most frequently used apps were in the category of drug dosing tools and patient diagnostic tools, with Medscape® and EMGuidance® being the most used apps. The most common criteria reported for recognizing a high-quality mHealth app were app updated regularly, scientifically evaluated, peer-reviewed and approved by a regulatory body. Improved awareness of the regulations pertaining to the use of mHealth apps amongst doctors is required.
